# Simultaneous detection of respiratory syncytial virus and human metapneumovirus by one-step multiplex real-time RT-PCR in patients with respiratory symptoms

**DOI:** 10.1186/s12887-017-0843-7

**Published:** 2017-03-27

**Authors:** Huey-Ling You, Shun-Jen Chang, Hong-Ren Yu, Chia-Chin Li, Chang-Han Chen, Wei-Ting Liao

**Affiliations:** 1 Departments of Laboratory Medicine, Kaohsiung Chang Gung Memorial Hospital, Kaohsiung, Taiwan; 2 Department of Medical Laboratory Sciences and Biotechnology, Fooyin University, Kaohsiung, Taiwan; 3 Department of Kinesiology, Health and Leisure Studies, National University of Kaohsiung, Kaohsiung, Taiwan; 4 Department of Pediatrics, Kaohsiung Chang Gung Memorial Hospital and Chang Gung University College of Medicine, Kaohsiung, Taiwan; 5 Institute for Translational Research in Biomedicine, Kaohsiung Chang Gung Memorial Hospital, Kaohsiung, Taiwan; 6 Institute for Translational Research in Biomedicine, Kaohsiung Chang Gung Memorial Hospital, Kaohsiung, Taiwan; 7 Department of Applied Chemistry, and Graduate Institute of Biomedicine and Biomedical Technology, National Chi Nan University, Nanto, Taiwan; 8 Department of Biotechnology, College of Life Science, Kaohsiung Medical University, No. 100 Shih-Chuan 1st Road, Kaohsiung, 807 Taiwan; 9 Department of Biological Sciences, National Sun Yat-Sen University, Kaohsiung, Taiwan

**Keywords:** RSV, hMPV, Real-time RT-PCR, Respiratory tract infection

## Abstract

**Background:**

Both respiratory syncytial virus (RSV) and human metapneumovirus (hMPV) are important viral pathogens causing respiratory tract infection (RTI) in the pediatric population. However, the clinical manifestations of RSV and hMPV infections are similar. Therefore, a reliable and rapid diagnostic tool is needed for diagnostic performance.

**Methods:**

In order to optimize diagnosis efficiency of RTI, the aim of this study is to establish a rapid and advanced method for simultaneous detecting RSV and hMPV in nasopharyngeal aspirates specimens from patients. We designed a one-step triplex real-time RT-PCR (qRT-PCR) protocol using TaqMan probes for detecting RSV and hMPV. The plasmid clones containing RSV nucleoprotein gene and hMPV fusion gene were established as reference standards. We used virus culture supernatants from 86 known pediatric RTI patient to test the specificity and sensitivity of our assay. Then we used total 222 nasopharyngeal aspirates specimens from pediatric patients hospitalized with respiratory symptoms to evaluate our assay.

**Results:**

Our one-step triplex qRT-PCR assay showed 100% sensitivity and specificity in testing RSV and hMPV in 86 known virus culture supernatants, with excellent linearity (R^2^ > 0.99) and reliable reproducibility (CV lower than 1.04%). This assay has a wide dynamic range 10^2^-10^9^copies/reaction (limit of detection; LOD = 100 copies/reaction). A total of 222 patients hospitalized with respiratory symptoms were enrolled for clinical evaluation. In these samples, our qRT-PCR assay detected 68 RSV positive and 18 hMPV positive cases. However, standard virus culture only detected 8 RSV positive cases and 0 hMPV cases. Based on this improved triplex qRT-PCR assay, we found that RSV infection was associated with severe inflammation by chest X-ray and occurrence of pneumonia which were not observed previously.

**Conclusions:**

In summary, we have developed a highly specific and sensitive one-step triplex qRT-PCR assay to detect hMPV and RSV simultaneously. This assay offers a valuable tool for routine diagnosis.

## Background

Acute respiratory tract infection (RTI) is estimated to be the second important cause of death throughout the world among children less than 5 years old [1]. Both respiratory syncytial virus (RSV) and human metapneumovirus (hMPV) are important viral pathogens inducing RTI [2–4]. RSV and hMPV are non-segmented, negative-strand, enveloped RNA viruses. Both of them are classified within the Pneumovirinae subfamily of the Paramyxoviridae family. RSV is implicated in the majority of respiratory tract infection, which accounts for 60–80% of the bronchiolitis cases in children below 12 months of age [5]. Recently, it has been demonstrated that hMPV also causes acute respiratory tract infections, similar to RSV [1, 6]. hMPV was first identified in 2001 from a pediatric patient with respiratory diseases in the Netherlands [7]. hMPV causes a wide spectrum of disease ranging from mild upper RTI to more severe lower RTI such as bronchiolitis or pneumonia. Second to RSV, hMPV causes bronchiolitis and accounts for 5–15% of child hospitalizations for RTI [8, 9].

Both RSV and hMPV infections induce disruption of respiratory epithelial architecture, sloughing of epithelial cells, loss of cilation and acute pulmonary inflammation characterized by alveolitis, interstitial inflammation and peribronchiolitis [7, 9]. Following, acute otitis media is the most common complication of RTI due to RSV or hMPV [8]. At present, the initial clinical manifestations of RSV are indistinguishable from those of hMPV [10–12]. One possible reference to distinguish RSV and hMPV infections is based on the epidemiological data. The median age of hMPV-positive hospitalized patients is 6-12 months, which is significantly higher than that of RSV (2-3 months) [13].

RSV and hMPV do not appear to be significantly associated with asymptomatic carriage in the respiratory tract of healthy persons. However, the diagnosis efficiency for these 2 virus infection is low using the classical virus culture methods. Both viruses are labile in the environment and are susceptible to drying [14]. For viral culture, RSV can growth in the HEp-2, A549, or Vero cells and usually takes 3–5 days to revealing characteristic syncytial cytopathic pattern [7, 15–17]. In contrast, hMPV can growth in the LLC-MK2 cells. The growth of hMPV is slow and often requires several blind passages before any cytopathic effect (CPE) is apparent, particularly following primary isolation, which often takes more than 10 days [18]. These lengthy viral culture processes, in part, limited the opportunity for optimal initial diagnosis and the following therapeutic adjustment for RTI patient. Therefore, a reliable and rapid diagnostic tool is needed. Rapid and accurate laboratory diagnosis of viral RTI is crucial for optimal clinical management, diminishes unnecessary use of antibiotics, and allows for use of antivirals when appropriate.

## Objectives

The aim of this study is to design and assess the diagnostic performance of clinical specimens for the simultaneous identification of RSV and hMPV by using one-step triplex qRT-PCR assay with TaqMan probes. To this aim, we first performed our primer and probe design. Then we used cultured viruses to evaluate the specificity of our primers using qRT-PCR. Finally, we used this established qRT-PCR assay to detect the viral load in nasopharyngeal aspirates.

## Methods

### Primers and probe design

The primers and probes for RSV, hMPV and GAPDH were designed by Primer 3 website (http://bioinfo.ut.ee/primer3-0.4.0/primer3/). We selected RSV nucleoprotein gene conserved regions as a target for our RSV primer and probe designs. For hMPV, we selected hMPV fusion protein gene conserved regions as a target for our primer and probe designs. The target sequence of our primer and probe designs was showed in Table 1. The specificity of these primer and probe sequences were tested by the BLAST algorithm.

**Table 1:** Design of the one-step triplex qRT-PCR assays for the detection of RSV, hMPV and Internal Control

Virus	Target gene	Name of primer or probe	Sequence (5′ → 3′)	Position
RSV	Nucleoprotein	RSV-F	TGATACACTSAACAAAGATCAACTTCTG	27–54^a^
		RSV-R	TCTCCTGTGCTMCGTTGRAT	73–92^a^
		RSV-probe	VIC-CATCCAGCAAATACAC-MGBNFQ	56–71^a^
hMPV	Fusion protein	hMPV-F	TCAGAATGCAGGGTCAACTGTT	156–177^b^
		hMPV-R	GACATGGTCTCCTCTTGTTTCACA	199–222^b^
		hMPV-probe	FAM-CAAGCTTCCCGTTCTCAGCC-MGBNFQ	179–197^b^
Internal Control	GAPDH	GAPDH-F	GAAGGTGAAGGTCGGAGTC	108–126^c^
		GAPDH-R	GAAGATGGTGATGGGATTTC	314–333^c^
		GAPDH-probe	NED-CAAGCTTCCCGTTCTCAGCC-MGBNFQ	285–304^c^

^a^GenBank Accession no. DQ780565.1

^b^GenBank Accession no. AY295956.1

^c^GenBank Accession no. NM_002046

To rapidly and accurately quantify the RSV and hMPV in nasopharyngeal aspirates, we combined 3 single qRT-PCR, amplifying RSV, hMPV and GAPDH (internal control), in a one-tube reaction. For this one-tube reaction, the probe for RSV was labeled with the dye VIC dye at its 5′ end and minor groove binder (MGB) at its 3′end. The probe for hMPV was labeled with the reporter 6-carboxyfluorescein (FAM) dye at its 5′ end and MGB at its 3′ end. The probe for GAPDH was labeled with NED dye at its 5′ end and MGB at its 3′ end. RSV, hMPV and GAPDH amplification signals were separately detected by the VIC, FAM and NED channels by multi-channel qRT-PCR detection system. The primers for direct sequencing as described following: hMPV-F: 5′-CAACTGTTTACTACCCAAATGA-3′, hMPV-R: 5′-ATAGGGTGTCTTCCTGTGC-3′; RSV-F: 5′-TTAACCAGCAAAGTGTTAGAYCTCAA-3′, RSV-R: 5′-CTGRTCATTTGTTATRGGCATATCATTG-3′.

### Virus culture and immunofluorescence assay

In brief, sterile beads were added to the samples, vortexed and processed routinely followed by centrifuged at 2000 rpm for 7 min. Specimens were then decontaminated by adding a 10% antibiotic mixture (Gibco, N.Y.USA) and incubating for 1 h at 4 °C. A volume of 200 μL of sample was inoculated into each of the following cell lines: MRC-5, HEp2, RD, MDCK, and LLCMK2 (ATCC, Manassas, VA, USA). One μΛ of maintenance medium (minimal essential medium with 1% fetal bovine serum) (Gibco, N.Y.USA) was added to each cell line and incubated at 37 °C for 14 days. The CPE was observed every other working day by inverted light microscopy (Olympus, Japan). After CPE appeared, cell smears were prepared and fixed in chilled acetone at −20 °C for 10 min and then tested by fluorescein-conjugated monoclonal antibody in a direct immunofluorescence assay (D^3^ Ultra 8^TM^ DFA Respiratory Virus Screening & ID Kit, Diagnostic Hybrids, USA). Stained cell smears were examined in a fluorescence microscope at 400× magnification (Olympus, Japan). Un-inoculated cell smear was used as negative control.

### Reference standard

To establish reference standards for RSV and hMPV viral load, we established 2 plasmid clones containing RSV nucleoprotein gene sequence and hMPV fusion protein gene sequence. These plasmids were amplified and cloned into a pCR® 2.1-TOPO® cloning vector using a TOPO TA cloning kit for sequencing (Invitrogen, Carlsbad, CA). Sequencing of the cloned insert established the fidelity of the sequence. Plasmid DNA containing each fragment sequence was purified, linearized by restriction enzyme digestion. The concentration of DNA was measured at least four times by a spectrophotometer (IMPLEN NanoPhotometer™, CA, USA), and the numbers of copies per μL were calculated using the mean values and the following formula: [(g/μL DNA)/(length × 660)] × 6.022 × 10^23^, where the length is the number of nucleotides. Tenfold dilutions equivalent to 10^2^ to 10^9^ copies/reaction of plasmid were used to determine the limit of detection (LOD) of the one-step triplex qRT-PCR assay.

### Sample collection

This study included infants (<24 months) and young children (<6 years old) admitted to the pediatric department of Kaohsiung Chang Gung Memorial Hospital. Children and infants were eligible if identified as having one or more of the following symptoms: difficulty in breathing, nasal discharge, blocked nose, cough, or fast breathing for age. Nasopharyngeal aspirates were prospectively collected from November, 2010 to August, 2011. Informed consent was signed from the parent/guardian of each child. The nasopharyngeal aspirates (NPAs) were sent for virus isolation and identification. The RSV and hMPV were identified by using two methods in each individual sample. One was one-step triplex qRT-PCR, the other was immunofluorescence approach to confirm the virus etiology after CPE formation. Ethical approval for the study was obtained from the Chang Gung Memorial Hospital Ethics Committee (99-2746B).

### Nucleic acid extraction and triplex RT-PCR (qRT-PCR)

RNA was extracted from the supernatants of cultured viruses or nasopharyngeal aspirates by QIAamp viral RNA mini kit (Qiagen, Valencia, CA, USA) according to the manufactures instructions. All the RNA extraction procedure was conducted at BSL-2 laboratory. The qRT-PCR of a final concentration of 12.5 μL Master Mix and 0.625 μL Enzyme Mix (TaqManR RNA-to-CT™ *1-Step* Kit, Applied Biosystems), 400 nM each primer; 200 nM each probe, plus 7 μL of target RNA and was made up to a volume of 25 μL with nuclease free water (Promega Corp. Madison, USA). The reactions were incubated at 48 °C for 30 min, followed by 95 °C for 15 s (inactivation reverse transcriptase/activation Taq polymerase), 40 cycles of 95 °C for 15 s (denaturation), and 54 °C for 1 min (annealing). RT-PCR assays were performed on an ABI 7500-fast (Applied Biosystem). If Ct >40 with lower concentration of virus, the amount of specimens was augmented for increasing the concentration of RNA and following One-step RT-PCR and direct sequencing.

### Statistic analysis

The sensitivity and specificity of triplex qRT-PCR results were analyzed by the receiver operating characteristic (ROC) method by SPSS software (SPSS statistical package version 17, SPSS Inc., Chicago, IL). The linearity and reproducibility for RSV and hMPV triplex qRT-PCR were analyzed by and linear regression and absolute quantification analysis (StepOnePlus software v2.3) provided by ABI 7500-fast PCR machine. Demographic data was analyzed by Student’s “t” test and chi-square using SPSS software. A *p*-value < 0.05 was considered significant.

## Results

### Assay specificity

A total of 86 virus culture stocks were used in specificity testing. The different virus etiology including RSV (*n =* 20), hMPV (*n =* 23), adenovirus (*n =* 10), enterovirus (*n =* 8), influenza A (*n =* 4), influenza B (*n =* 4), CMV (*n =* 5), HSV-1 (*n =* 3), parainfluenza type 1 (*n =* 3), parainfluenza type 2 (*n =* 3) and parainfluenza type 3(*n =* 3). No positive result was obtained for non-RSV or non-hMPV. We also used DEPC water as a negative control. The internal control and positive-control reactions were positive. Results showed that this one-step triplex qRT-PCR assay was specific and did not cross among RSV, hMPV and internal control GAPHD, with each other. Primer pair RSVF/RSVR and probe RSV-probe reacted only with RSV samples, whereas primers hMPVF/hMPVR and probe hMPV-probe allowed for the specific detection of hMPV isolates. The sensitivity and specificity of the one-step triplex qRT-PCR assay were both 100% when testing known viral stocks (Table 2).

**Table 2:** Analytical sensitivity and specificity of triplex qRT-PCR assay in 86 known virus culture supernatants

Virus Culture Stocks	Cultural Results No.(n)	Triplex qRT-PCR Results (n)	
		RSV+	hMPV+
RSV	20	20	0
hMPV	23	0	23
Adenovirus	10	0	0
Enterovirus	8	0	0
Influenza A virus	4	0	0
Influenza B virus	4	0	0
CMV	5	0	0
HSV-1	3	0	0
Parainfluenza virus 1	3	0	0
Parainfluenza virus 2	3	0	0
Parainfluenza virus 3	3	0	0
Total	86		

### Assay linearity

**Fig. 1:**
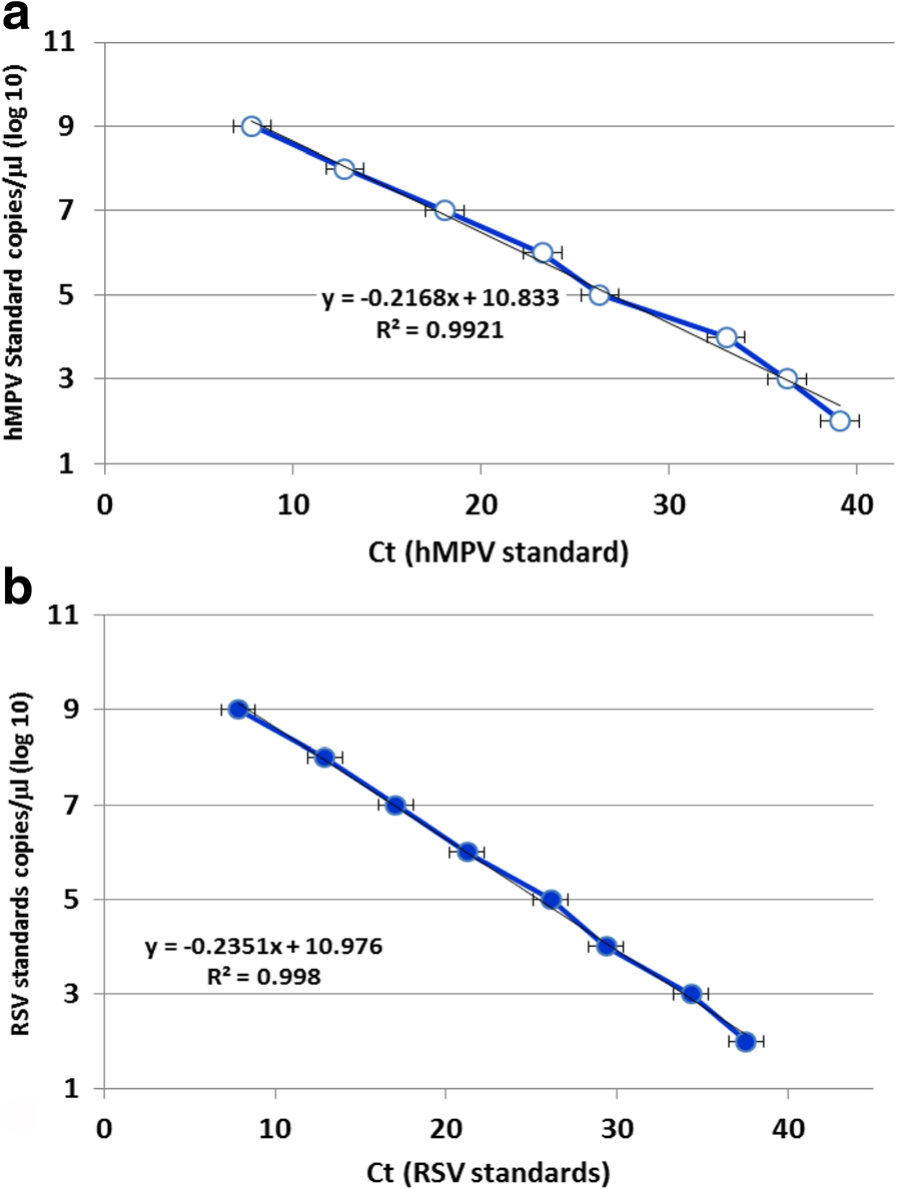
Analytical sensitivity of triplex qRT-PCR assay. **a** hMPV and (**b**) RSV qRT-PCR assay. Fluorescence development was detected via real-time detection in triplex qRT-PCR run by using a dilution range of 10^9^–10^2^ DNA molecules/reaction of the hMPV and RSV molecular standards (Graph generated by ESE quant tube scanner studio software)

Linearity of the one-step triplex qRT-PCR assay was determined using serial 10-fold dilutions of RSV and hMPV standard in viral transport medium (VTM) at the following concentrations:10^2^, 10^3^, 10^4^, 10^5^, 10^6^, 10^7^, 10^8^and 10^9^ copies/reaction. The extracted DNA was then analyzed by the triplex qRT-PCR assay. Three replicates were tested in a single run at each concentration. The relationship between the observed values and true concentrations of analyses was examined through linear regression. The limit of detection (LOD) of the triplex qRT-PCR assay was 100 copies/reaction. Linear regression analysis of the Ct values against the log10 RSV and hMPV plasmid concentration yielded R^2^ = 0.998 (Fig. 1a) and 0.992 (Fig. 1b), respectively. The detail statistic results were showed in Table 3.

**Table 3:** The linearity data for RSV and hMPV triplex qRT-PCR

Target	Slope	Intercept	r^2^	Linear range (copies/reaction)	LOD^a^ (copies/ reaction)
hMPV	−4.57	45.19	0.992	10^2^–10^9^	100
RSV	−3.91	44.85	0.998	10^2^–10^9^	100

^a^Limit of detection

### Assay precision

Precision was estimated by performing the one-step triplex qRT-PCR assay twice per day (each run separated by a minimum of 2 h) over 2 days, by the same technician using the same equipment and reagent lot numbers. The intra-assay variation was assessed by testing 2 samples with different viral loads (10^8^ and 10^2^ copies/reaction) 3 times in a single run, while the inter-assay variation was assessed by testing the same samples 3 times in 2 separate runs. In our assay, the coefficient of variation (CV, express imprecision) was lower than 5%. The intra-assay CV ranged from 0.10 to 0.47%, while the inter-assay CV ranged from 0.23 to 1.04% (Table 4).

**Table 4:** Reproducibility of the triplex qRT-PCR assay

	Target	Conc. (copies/reaction)	Number of determinations	Mean Ct	SD	CV (%)
Intra-assay	hMPV	10^8^	3	13.90	0.033	0.24
		10^2^	3	35.69	0.170	0.47
	RSV	10^8^	3	15.80	0.015	0.10
		10^2^	3	39.10	0.112	0.29
Inter-assay	hMPV	10^8^	6	13.89	0.032	0.23
		10^2^	6	36.46	0.863	0.91
	RSV	10^8^	6	16.18	0.430	1.04
		10^2^	6	38.98	0.225	0.58

### Clinical evaluation

**Fig. 2:**
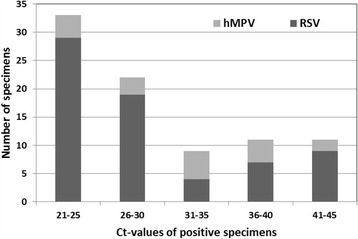
Ct values distribution of RSV and hMPV positive clinical specimens. Our one-step triplex qRT-PCR results showed that the viral load of the RSV or hMPV-positive samples from clinical specimens varied over a wide range, presenting threshold cycles between 21 and 44. RSV has a maximum occurrence detected between Ct 21 and 25, but hMPV has a maximum occurrence between Ct 31 and 35

A total of 222 pediatric patients hospitalized with respiratory symptoms were enrolled from November 2010 through August 2011. Study patients were 61% male and 39% female with a mean age of 0.91 years old (ranges from32 days to 14 years old). The admitting diagnoses of these infants were bronchiolitis (58%), brochopneumonia (24%), and pneumonia (18%). Our one-step triplex qRT-PCR results showed that the viral load of the RSV or hMPV-positive samples from clinical specimens varied over a wide range, presenting threshold between Ct 21 and 44 (21 and 44 cycles). As a result, 68 specimens (30.6%) were found RSV positive, 18 specimens (8.1%) were found hMPV positive by our one-step triplex qRT-PCR. However, standard virus culture only detected 8 RSV positive cases (3.6%) and 0 hMPV cases (0%) (Table 5). In detecting RSV, the major distribution of threshold cycles ranged between Ct 21 and 25 (1,035,656 copies/ reaction ~77,992 copies/reaction). In detecting hMPV, the major distribution of threshold cycles ranged between Ct 31 and 35 (8395 copies/ reaction ~476 copies/ reaction) (Fig. 2).

**Table 5:** Comparing of triplex qRT-PCR assay and virus culture in nasopharyngeal aspirates from 222 patients hospitalized with respiratory symptoms

Virus Culture Stocks	Cultural Results No.(n)	Triplex qRT-PCR Results (n)	
		RSV+	hMPV+
RSV	8	8	0
hMPV	0	0	0
Adenovirus	16	0	0
Enterovirus	8	0	0
Influenza A virus	1	0	0
Influenza B virus	1	0	0
CMV	3	0	0
Parainfluenza virus 1	3	0	0
Parainfluenza virus 2	1	0	0
Parainfluenza virus 3	13	0	0
^a^NVI	168	60	18
Total	222	68	18

^a^ *NVI* Non virus identified using virus culture

### qRT-PCR RSV positive patients were associated with increased severe inflammation and pneumonia

The demographic data based on RSV/hMPV one-step triplex qRT-PCR detection in 222 infants hospitalized with respiratory symptoms showed that male patients have higher percentages in RSV or hMPV infection. RSV and hMPV infection cases were significantly associated with severe lung inflammation based on chest X-Ray index. Complication of pneumonia was observed in more than 90% of RSV or hMPV infection patient (Table 6). In addition, we found that RSV or hMPV infection is not associated with C-reactive protein level, indicating the importance of making definite diagnosis in early intervention of RSV and hMPV associated adverse effects, such as severe inflammation and pneumonia.

**Table 6:** Demographic and clinical characteristics from 222 patients hospitalized with respiratory symptoms

	Triplex qRT-PCR Results			*P*-value
	Negative(*n =* 136)	RSV+ (*n =* 68)	hMPV+ (*n =* 18)	
Gender (%)				
F	62(45.6)	22(32.4)	3(16.7)	0.030*
M	74(54.4)	46(67.6)	15(83.3)	
Age (years-old)				
	1.38 ± 1.89	1.15 ± 1.22	1.00 ± 1.29	0.119
CXR index (%)				
N/A	27(19.9)	3(4.4)	4(22.2)	0.008*
Mild inflammation	43(31.6	18(26.5)	4(22.2)	
Severe inflammation	66(48.5)	47(69.1)	10(55.6)	
ICU treatment (%)				
No	77(56.6)	47(69.1)	12(66.7)	0.118
Yes	59(43.4)	21(30.9)	6(33.3)	
Pneumonia (%)				
No	95(69.9)	6(8.8)	1(5.6)	0.001*
Yes	41(30.1)	62(91.2)	17(94.4)	
Hypersomina (%)				
No	131(96.3)	65(95.6)	18(100)	0.621
Yes	5(3.7)	3(4.4)	0(0)	
Intubation (%)				
No	134(98.5)	67(98.5)	17(94.4)	0.599
Yes	2(1.5)	1(1.5)	1(5.6)	
CRP index (%)				
N/A	8(5.9)	3(4.4)	0(0)	0.938
< 5	62(45.6)	34(50.5)	6(33.3)	
Positive	66(48.5)	31(45.6)	12(66.7)	

*N/A* not available, *CXR index* Chest X-Ray index, *ICU* Intensive care unit, *CRP* C-reactive protein

*P < 0.05

## Discussion

In this study, we established a rapid and internally controlled triplex qRT-PCR assay that can identify RSV and hMPV virus in one reaction mixtures. The validation parameters described here were in accordance with procedural and statistical methods as recommended in the Validation of Analytical Procedures (Clinical and laboratory standard institute, CLSI 2009). The development of fluorescent methods and instruments that allow real-time monitoring of the amplification process is considered an important step for molecular biology [19]. Real-time RT-PCR has become a well-established procedure in terms of rapid detection of nucleic acid targets. Incorporation of housekeeping genes as internal control is important to exclude false negative results due to incomplete RNA extraction or inhibition of PCR. The optimized assays reported here, allow specific and sensitive detection of RSV and hMPV, as well as of an internal amplification control. Furthermore, this method can be multiplexed using different fluorogenic dyes for the three probes.

In this current study, we determined the copy numbers of RSV and hMPV and internal control (GAPDH) at the same time in one test sample. The procedures of RNA extraction and reagent preparation and efficiency of instruments could be monitor by triplex qRT-PCR in one sample. Furthermore, this approach also reduced the results of “false-negative” and cost down. However, some groups detected target virus and internal control by using duplex qRT-PCR that may increase the time-consuming [20].

This triplex qRT-PCR provides several clinical advantages. First, this method saves the time for diagnostic performance, compared to virus culture. Second, our method increased the accuracy of RSV and hMPV detection. The rapid and accurate RSV and hMPV detection may help for early intervention of the related clinical events or complications, such as severe lung inflammation and pneumonia.

## Conclusion

Altogether, the three sets of primer/probe in the one-step triplex qRT-PCR assay presented in this study is a specific and sensitive diagnostic tool to rapid screening for the detection of RSV and hMPV RNA. It is useful method in routine laboratory diagnostics.

## Abbreviations

CPE: Cytopathic effect
hMPV: Human metapneumovirus
LOD: Limit of detection
NPAs: Nasopharyngeal aspirates
RSV: Respiratory syncytial virus
RTI: Respiratory tract infection

## References

[1] Paget SP, Andresen DN, Kesson AM, Egan JR (2011). Comparison of human metapneumovirus and respiratory syncytial virus in children admitted to a paediatric intensive care unit. J Paediatr Child Health.

[2] Berkley JA, Munywoki P, Ngama M, Kazungu S, Abwao J, Bett A, Lassauniere R, Kresfelder T, Cane PA, Venter M (2010). Viral etiology of severe pneumonia among Kenyan infants and children. JAMA.

[3] Nair H, Nokes DJ, Gessner BD, Dherani M, Madhi SA, Singleton RJ, O’Brien KL, Roca A, Wright PF, Bruce N (2010). Global burden of acute lower respiratory infections due to respiratory syncytial virus in young children: a systematic review and meta-analysis. Lancet.

[4] Forgie IM, O’Neill KP, Lloyd-Evans N, Leinonen M, Campbell H, Whittle HC, Greenwood BM (1991). Etiology of acute lower respiratory tract infections in Gambian children: I. Acute lower respiratory tract infections in infants presenting at the hospital. Pediatr Infect Dis J.

[5] Nagakumar P, Doull I (2012). Current therapy for bronchiolitis. Arch Dis Child.

[6] Manoha C, Espinosa S, Aho SL, Huet F, Pothier P (2007). Epidemiological and clinical features of hMPV, RSV and RVs infections in young children. J Clin Virol.

[7] van den Hoogen BG, de Jong JC, Groen J, Kuiken T, de Groot R, Fouchier RA, Osterhaus AD (2001). A newly discovered human pneumovirus isolated from young children with respiratory tract disease. Nat Med.

[8] Papenburg J, Boivin G (2010). The distinguishing features of human metapneumovirus and respiratory syncytial virus. Rev Med Virol.

[9] van den Hoogen BG, Osterhaus DM, Fouchier RA (2004). Clinical impact and diagnosis of human metapneumovirus infection. Pediatr Infect Dis J.

[10] Schildgen V, van den Hoogen B, Fouchier R, Tripp RA, Alvarez R, Manoha C, Williams J, Schildgen O (2011). Human Metapneumovirus: lessons learned over the first decade. Clin Microbiol Rev.

[11] Louie JK, Schnurr DP, Pan CY, Kiang D, Carter C, Tougaw S, Ventura J, Norman A, Belmusto V, Rosenberg J (2007). A summer outbreak of human metapneumovirus infection in a long-term-care facility. J Infect Dis.

[12] Wilkesmann A, Schildgen O, Eis-Hubinger AM, Geikowski T, Glatzel T, Lentze MJ, Bode U, Simon A (2006). Human metapneumovirus infections cause similar symptoms and clinical severity as respiratory syncytial virus infections. Eur J Pediatr.

[13] Kahn JS (2006). Epidemiology of human metapneumovirus. Clin Microbiol Rev.

[14] Muller A, Tillmann RL, Muller A, Simon A, Schildgen O (2008). Stability of human metapneumovirus and human coronavirus NL63 on medical instruments and in the patient environment. J Hosp Infect.

[15] Peret TC, Boivin G, Li Y, Couillard M, Humphrey C, Osterhaus AD, Erdman DD, Anderson LJ (2002). Characterization of human metapneumoviruses isolated from patients in North America. J Infect Dis.

[16] Boivin G, Abed Y, Pelletier G, Ruel L, Moisan D, Cote S, Peret TC, Erdman DD, Anderson LJ (2002). Virological features and clinical manifestations associated with human metapneumovirus: a new paramyxovirus responsible for acute respiratory-tract infections in all age groups. J Infect Dis.

[17] Williams JV, Harris PA, Tollefson SJ, Halburnt-Rush LL, Pingsterhaus JM, Edwards KM, Wright PF, Crowe JE (2004). Human metapneumovirus and lower respiratory tract disease in otherwise healthy infants and children. N Engl J Med.

[18] Zhang Y, Wei Y, Li J, Li J (2012). Development and optimization of a direct plaque assay for human and avian metapneumoviruses. J Virol Methods.

[19] Holland PM, Abramson RD, Watson R, Gelfand DH (1991). Detection of specific polymerase chain reaction product by utilizing the 5′----3′ exonuclease activity of Thermus aquaticus DNA polymerase. Proc Natl Acad Sci U S A.

[20] Jokela P, Piiparinen H, Luiro K, Lappalainen M (2010). Detection of human metapneumovirus and respiratory syncytial virus by duplex real-time RT-PCR assay in comparison with direct fluorescent assay. Clin Microbiol Infect.

